# Understanding reasons for and strategic responses to administrative health data misreporting in an Indian state

**DOI:** 10.1093/heapol/czac065

**Published:** 2022-08-09

**Authors:** Ankita Meghani, Daniela C Rodríguez, David H Peters, Sara Bennett

**Affiliations:** Department of International Health, Johns Hopkins Bloomberg School of Public Health, 615 N Wolfe St, Baltimore, MD 21205, USA; Department of International Health, Johns Hopkins Bloomberg School of Public Health, 615 N Wolfe St, Baltimore, MD 21205, USA; Department of International Health, Johns Hopkins Bloomberg School of Public Health, 615 N Wolfe St, Baltimore, MD 21205, USA; Department of International Health, Johns Hopkins Bloomberg School of Public Health, 615 N Wolfe St, Baltimore, MD 21205, USA

**Keywords:** Data quality, misreporting, data manipulation, health management information systems, administrative data, India

## Abstract

The misreporting of administrative health data creates an inequitable distribution of scarce health resources and weakens transparency and accountability within health systems. In the mid-2010s, an Indian state introduced a district ranking system to monitor the monthly performance of health programmes alongside a set of data quality initiatives. However, questions remain about the role of data manipulation in compromising the accuracy of data available for decision-making. We used qualitative approaches to examine the opportunities, pressures and rationalization of potential data manipulation. Using purposive sampling, we interviewed 48 district-level respondents from high-, middle- and low-ranked districts and 35 division- and state-level officials, all of whom had data-related or programme monitoring responsibilities. Additionally, we observed 14 district-level meetings where administrative data were reviewed. District respondents reported that the quality of administrative data was sometimes compromised to achieve top district rankings. The pressure to exaggerate progress was a symptom of the broader system for assessing health performance that was often viewed as punitive and where district- and state-level superiors were viewed as having limited ability to ensure accountability for data quality. However, district respondents described being held accountable for results despite lacking the adequate capacity to deliver on them. Many rationalized data manipulation to cope with high pressures, to safeguard their jobs and, in some cases, for personal financial gain. Moreover, because data manipulation was viewed as a socially acceptable practice, ethical arguments against it were less effective. Potential entry points to mitigate data manipulation include (1) changing the incentive structures to place equal emphasis on the quality of data informing the performance data (e.g. district rankings), (2) strengthening checks and balances to reinforce the integrity of data-related processes within districts and (3) implementing policies to make data manipulation an unacceptable anomaly rather than a norm.

Key messages

- Opportunities for data manipulation were created when local staff had limited discretion over data-related activities and the leadership expressed little demand for accurate data and weakly enforced accountability mechanisms for data quality.
- High performance pressures for becoming a top-ranking district in the state over a short time period and a punitive work culture increased pressures to manipulate data. Social norms and the importance of maintaining job and financial security were also reasons used to rationalize data manipulation.
- Three entry points to mitigate data manipulation are as follows: (1) changing the incentive structures to ensure equal emphasis on data quality and on performance data; (2) strengthening checks and balances to reinforce the integrity of data quality processes at all levels and (3) implementing system-wide policies that make data manipulation an unacceptable anomaly.

## Introduction

The Performance of Routine Information Systems framework identifies technical, organizational and behavioural determinants that are critical for improving the quality and use of the health management information system (HMIS) or administrative data for decision-making (Aqil *et al.*, 2009). While these determinants are useful for identifying interventions to improve administrative data quality and use, conceptual frameworks or theories that explain how and why administrative data are misreported or manipulated are lacking, yet are critically important in identifying how one might intervene to prevent these practices.

We define the manipulation of administrative health data or data manipulation as the fabrication or alteration of data, done with the aim of furthering one’s personal interests or coping with systemic pressures, for example, by giving falsely positive impressions of health sector achievements or hiding negative data. Data manipulation may involve the misuse of power by officials within the health system and can sometimes occur in clandestine ways. To better understand the forces that result in data manipulation and consider how best to respond to this practice in a health system, we adapted an existing theoretical framework that covers many of these concepts (Vian, 2008).

Data manipulation, sometimes referred to as data falsification or false reporting (Sahay *et al.*, 2017), is a widespread phenomenon across many settings and types of data. Public health researchers have observed such challenges in national statistics (Jerven, 2013; Sandefur and Glassman, 2015); they have reported on the falsification of administrative data (Qazi and Ali, 2009; Mercader *et al.*, 2017) and explored how perceptions of compromised data quality affect data use (Mutemwa, 2006; Setel *et al.*, 2007). The consequences of making decisions based on manipulated and poor quality data have been far-reaching, resulting in poor planning and inequitable distribution of resources and delivery of health services (Mackey *et al.*, 2018) and a breakdown in transparency and accountability processes within a health system (Transparency International, 2019). For these reasons, understanding the factors that influence the accuracy of HMIS data, the largest routinely collected data source about the health services delivered to a population, is of paramount importance.

To improve HMIS data quality and increase transparency in data reporting, many low- and middle-income countries have introduced district health information systems (DHIS2, 2019). These systems have facilitated the (1) replacement of paper-based reports with mobile health (mHealth) applications to collect data (Asangansi *et al.*, 2013; Biemba *et al.*, 2017), (2) automation of data validation processes to identify reporting errors (Ministry of Statistics and Program Implementation, Government of India, 2018; Burnett *et al.*, 2019) and (3) establishment of web-based data dashboards to allow real-time monitoring of health programmes (Nutley *et al.*, 2013; Mutale *et al.*, 2018).

While these technical advances in data collection have helped improve aspects of data management, quality and use, the challenge of subpar HMIS data quality persists (Kiberu *et al.*, 2014; Ndabarora *et al.*, 2014; Morton *et al.*, 2016; Phillips *et al.*, 2019). Quantitative studies have shown a systematic over-reporting of certain HMIS health indicators (Sharma *et al.*, 2016; Phillips *et al.*, 2019; Singh *et al.*), and qualitative studies have described how a combination of individual- and organizational-level factors, such as the lack of interest and ownership in the data (Hernández-Ávila *et al.*, 2013) and poor accountability processes in the health system, weakens the enforcement of HMIS data quality standards (Qazi and Ali, 2009; 2011; Ramesh *et al.*, 2012). A key question remains: why are data manipulated or misreported in the first place?

Answering this question requires unpacking contextual factors, organizational factors and interpersonal dynamics of actors who may be incentivized to allow data manipulation to thrive. In this paper, we first describe the types of data manipulation observed in an Indian state and then apply a theoretical framework originally developed to understand the factors affecting corruption (Vian, 2008), examining by turn (1) the pressures and (2) the opportunities for data manipulation and (3) the rationalization of data manipulation by those involved. A deeper understanding of these collective factors driving data manipulation may provide insights into how to address this problem.

## Methods

### Study context

Given the sensitive nature of this study and the confidential information our respondents revealed, we are anonymizing the state as well as the respondents’ district and division affiliations to protect their identities.

The state government implemented a series of initiatives in the mid-2010s to strengthen the processes for HMIS data quality and data use in decision-making at the district and state levels. These involved extensive investment in systems development, technology, training, supervision and support. First, the government implemented a new online HMIS to gather relevant data on health services being provided at the facility and community levels. Among the nearly 1000 data elements in the HMIS, some were used to calculate the monthly district rankings; others captured data on services like antenatal care and institutional deliveries that were incentivized by the national government (Government of India, 2018) and the remaining data elements were collected for routine monitoring.

Second, the government established data validation committees at two administrative levels below the state—at district and block levels—to ensure that accurate data were available for decision-making. The government also built automated data validation checks within the HMIS web-based portal to identify data errors and instituted data quality audits and supportive supervision visits at the district and block levels. Finally, to facilitate the use of these validated data for decision-making across the health system, the government developed a Health Dashboard that was accessible to health staff at the block, district and state levels. Populated using the HMIS data, the Dashboard ranked each district relative to others based on their performance on priority health indicators every month. The Dashboard also included two data quality metrics: the percent completeness of data elements being reported (i.e. percentage of missing values for indicators in the submitted monthly facility reports) and the percentage outliers in the data elements based on the facility-level HMIS reports (i.e. percentage of moderate or extreme outliers calculated for each indicator reported in the submitted monthly facility report). The policy did not outline any explicit awards or punishments for high- or low-ranking districts. However, the government expected that district ranking indicators would be examined during district-level programme review meetings to identify performance gaps and develop action plans to drive improvement. Given this context, the state offered an opportunity to investigate why data are manipulated or misreported despite the implementation of technical solutions to improve data quality.

### Conceptual framework

**Figure 1.**
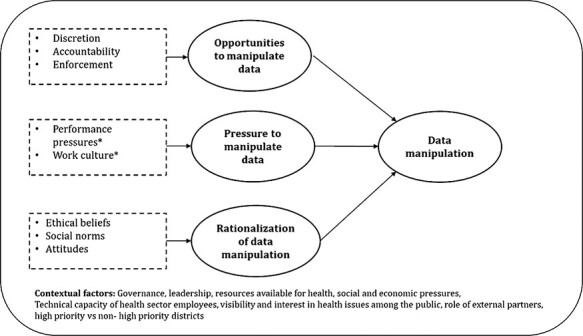
Conceptual framework for the study

Based on our formative research, we adapted a conceptual framework (Vian, 2008) to investigate key factors driving data manipulation in the state (Figure 1). To identify factors that created opportunities for data manipulation, first, we examined the level of discretion and autonomy of actors, the accountability mechanisms for performance and data quality and processes for detecting data manipulation and enforcing sanctions to curb future occurrences. Second, we studied pressures that incentivized data manipulation, e.g. performance pressures from one’s superiors and peers as well as pressures associated with the organizational work culture. Third, we explored the social norms, ethical beliefs and attitudes of health staff to understand how data manipulation was rationalized by those involved. Finally, acknowledging the importance of context, we remained open to studying how related health system factors, like availability of resources, workload, organizational culture and leadership styles, may influence data manipulation and its drivers.

*Denotes the adaptations to the original framework on corruption in the health sector developed by Vian (2008).

### Sampling

We conducted 83 in-depth interviews with government staff and leadership at the district, division and state levels. Following the principle of maximum variation sampling, we purposively selected 16 high-, middle- and low-ranked districts based on their rankings in the Health Dashboard. Within each district-ranking category, we selected a combination of both high-priority and non-high-priority districts (Government of India, 2015). To ensure a broad representation of district respondents, within each district, we purposively interviewed respondents who were involved in at least one data-related activity, such as data analysis, data validation or data review (e.g. reviewing data to monitor programme performance or make a programme-related decision) and included both government employees of the state’s Health Directorate and contractual employees of the National Health Mission (NHM) and state partners. Overall, we interviewed 48 district-level respondents as well as 35 division- and state-level officials in NHM and the Directorate responsible for monitoring district health programmes to understand the broader organizational context. Finally, we observed 14 district-level data validation and programme review meetings in eight of the 16 districts where we conducted interviews.

### Data collection

Our district-level interview guide aimed to elicit respondents’ perceptions on (1) the quality of existing administrative data; (2) current practices to promote data quality; (3) the prioritization of data quality initiatives within the health department; (4) individual, organizational and contextual factors that influence data quality processes and (5) potential opportunities to uphold data quality standards within the health system. Our division- and state-level guide focused on understanding which data are used for decision-making, how they are used, and potential challenges and opportunities for improving the use of data for decision-making in the state.

Interviews were conducted primarily in respondents’ offices in a local language or English depending on their preference. Before each interview, we obtained written informed consent. Interviews were audio-recorded except for 22 interviews when respondents preferred hand-written notes to be taken. Interviews generally lasted between 30 and 90 min. All audio-recorded interviews were transcribed verbatim and translated to English as needed by a qualified transcription agency; AM reviewed all transcripts for accuracy.

Interviews (Table 1) and meeting observations (Table 2) were conducted in three phases: December 2018; February–March 2019 and August–October 2019. During each round of data collection, the study team debriefed biweekly to discuss emerging findings, triangulate data by respondent type and identify probes for subsequent interviews. Between interview rounds, we also analysed the data to identify recurring themes, which informed the selection of topics during the subsequent interview round. Our iterative approach to data collection and analysis was important for reaching saturation (Morse, 2015).

**Table 1.** Types of positions held by respondents in the Indian state

Level	Type of position	Description	Number of respondents
District	Administrative officials	District magistrates and chief development officers	2
	Health officials	Chief medical officers	2
	District staff	Programme staff^a^	46
		Data staff^b^	
Division		Monitoring and evaluation specialists	5
State		Directorate: Directors and Joint Directors NHM: Programme managers and additional research officers	30
Total			83

a Responsible for managing and monitoring the implementation of health programmes; positions include assistant chief medical officers and district immunization officers.

b Responsible for collating, reviewing and analysing health programme data; positions include assistant research officers, district programme managers, district data managers, data entry operators, monitoring and evaluation specialists.

**Table 2.** Meeting observed at the district level in the Indian state

Meeting types	Number of observations
Data validation committee meetings	6
Programme review meetings	8
Total	14

### Analysis

We conducted a thematic analysis using the framework method (Gale *et al.*, 2013). First, we inductively coded 20 transcripts line-by-line and used the conceptual framework categories to develop an analytical framework that captured data on (1) the rationalization of data manipulation; (2) opportunities for data manipulation; (3) pressures and work-related stressors and (4) other factors. The detailed sub-categories in the analytical framework are in the Appendix. We extracted data from the interview transcripts, notes and meeting observations into a spreadsheet with the analytical framework. Then, memos for each category were prepared, summarizing the overall findings, identifying deviant cases, comparing and contrasting potential conflicting findings reported by respondents (Boeije, 2002) and triangulating meeting observations with interview findings (Carter *et al.*, 2014). These approaches highlighted areas of convergence and helped generate a comprehensive account of data manipulation. Memos were shared with team members to deepen the discussion and understanding of the data. After the analysis, a meeting was conducted with a couple of respondents to solicit their views on the findings as a way of member checking.

## Results

Despite recent improvements in data quality, district-level respondents perceived that data manipulation and the pressures to manipulate data were common across both high-priority and non-high-priority districts. Therefore, we do not present our results by this stratification. Similarly, because monthly district rankings significantly fluctuated over the course of our study, we do not stratify the results by low-, middle- and high-performing districts. Below, we describe the types of data manipulation observed by respondents, the opportunities and pressures to change data and how these practices were rationalized.

### Types of data manipulation

District staff observed four types of data manipulation: (1) over-reporting data; (2) underreporting data; (3) retrofitting data to match inventory data and (4) hiding data (Table 3).

**Table 3.** Types of administrative data manipulation observed at the block and district levels in the Indian state

Type of data manipulation	How it works	Examples of indicators being manipulated
Over-reporting progress	The numerator, i.e. the number of services provided, reflects a longer time period (e.g. 45 days), while the denominator is assumed to be for the typical 30-day reporting period Data may be made up to reflect progress	Closely monitored district ranking indicators for priority programmes and national campaigns (e.g. the ratio of health worker incentives paid against total institutional deliveries) Indicators associated with financial incentives (e.g. institutional deliveries and bed occupancy rates) Indicators are difficult to cross-validate with other indicators (e.g. supplies distributed and community activities conducted)
Underreporting indicators	Reporting fewer incidents than actually occur	Indicators that may reflect poorly on health workers (e.g. maternal deaths)
Retrofitting service data to match inventory data	Data on the number of health commodities were calculated based on inventories left in the facility at the end of the month, rather than reflecting an actual count of commodities distributed by health workers	Data on health commodities, like oral contraceptive pills, and multi-dose vaccine vials
Hiding data	Data reflecting low progress on certain health indicators are not presented during review meetings with leadership	Information on low-performing indicators is removed from presentations with their superiors

District staff described over-reporting of positive health indicators that were closely monitored, for example, indicators in the district rankings, those associated with health campaigns or associated with financial incentives. Over-reporting involved inflating the number of services provided (numerator) over a fixed reporting period (denominator). According to district data staff, ranking indicators (e.g. the total number of institutional deliveries) were often over-reported to demonstrate higher than actual progress before meetings with district administrative officials. One data staff described the over-reporting of a ranked indicator that measured the timely disbursement of incentives to health workers, measured by the ratio of incentives paid out to health workers against the total number of institutional deliveries for which incentives should be given:

*To prepare for District Health Society meetings [with district administrative officials], I collect the data till 20th of the month. I see that the ASHA [frontline health workers] payments till 20^th^ is not so good, so they [chief medical officers] ask to change the data of ASHA payments [numerator] but the number of [institutional] deliveries should be same [denominator]* (I-5).

District staff observed similar practices at the block and community levels where data were over-reported by staff before monthly meetings with their supervisors:

*Our ANMs [health workers] say that this is the actual work done and this is the work that is remaining…even if 4 people have been vaccinated, they write 14 people vaccinated in the report. They say they will vaccinate the remaining 10 when they come next* (I-32, District programme staff).

While personal financial gain may influence community health workers to over-report incentivized indicators like childhood immunizations, district staff explained that those who manipulated the data were not always the direct beneficiaries of financial incentives. Their supervisors at the block level may over-report incentivized indicators and through complex kick-back systems receive a portion of the incentives given to community health workers.

District staff also described the underreporting of negative data that reflected poorly on a health worker’s performance. They felt this led to systematic underreporting of maternal and neonatal deaths, including stillbirths, which were reported as no deaths or fewer than actual deaths. This challenge was also broadly acknowledged by district administrative officials, who emphasized the importance of changing this practice:

*Staff are afraid to report a maternal death because they feel they will get some punishment or be blamed for not taking care of the mother. They escape by not reporting maternal or child deaths. If the message reaches a higher level, then an investigation may be conducted to find out the reason for death. If the HB [hemoglobin] was less, then superiors will ask ‘why was this not taken care of?’* (I-36).

Drawing on experiences from their supportive supervision visits at the block and community levels, district staff observed other subtle forms of data manipulation. They described how data on the distribution of health commodities (e.g. oral contraceptive pills) did not reflect the actual number of commodities distributed by health workers. Actually, they were retrofitted to match the existing health facility inventories and were not based on verifiable data reported in health workers’ registers. The data were calculated based on stocks or inventory left in the health facilities at the end of the month as compared with the beginning of the month.

Finally, district staff described requests by district health officials to hide data on poorly performing indicators from meeting presentations with their supervisors, such as district administrative officials, to avoid bringing attention to the low provision of health services or commodities in the district.

### Opportunities for data manipulation

#### Discretion

Many district staff observed that unchecked power and authority exercised by senior block and district health officials created opportunities for data manipulation. Their high degree of discretion coupled with little demand for accurate data resulted in low prioritization of data quality processes, which was problematic for two main reasons.

First, without explicit support from their health officials, data staff in blocks and districts were unable to convene data validation meetings and unable to create accountability for other health staff to deliver on their data quality-related tasks. Second, data staff’s lack of seniority limited their ability to prevent district and block health officials from requiring data manipulation. For example, when district health officials demanded that data should be fixed or changed, district data staff described their reluctance to push back because their job security was directly tied to district health officials’ opinion of them. Furthermore, the lack of avenues available to district staff, particularly data staff, to report their grievances was problematic; as one stated, ‘who would they raise their voices to when all the feedback goes back to one person?’ (I-16). Similar challenges were described at the block level between block staff and block health officials.

#### Accountability

District staff described the implementation of two competing forms of accountability within the health system: district leadership and state leadership created strong accountability mechanisms for performance, while weakly enforcing accountability for data quality. For accurate data to inform district rankings, the enforcement of both forms of accountability is needed.

##### Accountability for performance

Assessment of district performance was largely based on the monthly district rankings in the Health Dashboard. A district’s rank was routinely reviewed during meetings with district health and administrative officials. If the district’s ranking was low, district data staff felt the focus shifted to pinpointing blame rather than examining the drivers of low performance, as one stated:

*They [District administrative officials] just want to see their A grading of the district. No one wants to know that we cannot do well in the outpatient department because we do not have doctors* (I-32).

Following these meetings, district health and administrative officials reportedly sanctioned punishments via official letters or requests for ‘action taken reports’ requiring explanations for poor performance. Unofficial forms of communication like threats to withhold salaries or delay approval of holidays were also reportedly used by district leadership, a point corroborated by the district health officials and administrative leaders we interviewed and also noted in our meeting observations.

The district leadership’s emphasis on accountability for performance was seen to mirror state-level priorities, as meetings between districts and states predominantly focused on monitoring targets and performance based on the district rankings. Most state respondents corroborated this, saying that they used data to monitor performance as opposed to identify or address issues of data quality.

##### Accountability for data quality

Despite the presence of two formal mechanisms to improve data quality—supportive supervision visits and data validation committee meetings—six factors appeared to weaken their enforcement: (1) poor understanding of data quality, (2) high workload, (3) overemphasis on using data for ranking performance, (4) perception that district performance reflects one’s own performance, (5) systemic corruption and (6) weak processes for disciplining or dismissing staff.

First, district data staff described an inadequate level of understanding about data quality—what good data quality means and how it is measured—among district administrative and health officials. Often ‘good data’ were interpreted as ‘good performance’ and not necessarily ‘good quality data’. Some district and state respondents rationalized poor performance by explaining that services were being systematically under-reported:

*It is not that the doctors are not seeing the patients, day by day the OPD [Outpatient Department] load is increasing… but it is not reflected in the data* (I-58, State-level respondent).

This perception that services are being delivered but being underreported may also explain why reporting errors were not given due consideration when identified during district data validation meetings:

*They [data entry operators or community health workers] do not worry about writing the wrong data. They just say, ‘Oh, it went wrong, tell me what to fill here.’ Here, data does not mean true data* (I-18, District data staff).

Some district data staff further explained that less time was invested in recording, reviewing and assessing the data quality of indicators that did not inform the district-level rankings and were not associated with financial incentives.

Second, high workload associated with the implementation of a number of national health programmes and fortnightly health campaigns also contributed to the low prioritization of data quality accountability mechanisms, like supportive supervision. As one district data staff noted: ‘In practice, we have so much work that supportive supervision actually becomes too much…there is no time to actually do it’ (I-27). District data staff also reported that some of them preferred ‘changing the data’ rather than conducting supportive supervision (I-43). When supportive supervision did occur, district data staff questioned the quality of these visits, describing them as informal ‘tea-visits’ rather than official validations of web-based data (I-43). This laxness was attributed to district staff’s minimal training on using the supportive supervision checklist or lack of sincerity towards work.

Third, the state leadership’s emphasis on performance rankings over data quality contributed to the weak enforcement of data quality at the district and block levels. Several district data staff felt this was evident in the little attention given to data quality issues when data demonstrated good performance (e.g. a high district ranking): ‘If the performance is good on the basis of data, no one is going to ask anything [about data quality]’ (I-43). In a similar vein, one state-level respondent explained if the focus is on demonstrating improvement, using the data source that fits that messaging becomes the priority.

State-level respondents also said that there was limited demand for data quality from the state. According to some, the lack of consideration for data quality was perhaps most reflected in a decision to minimize the functions of a data unit within the Directorate. Until 2015, this cell was responsible for reviewing paper-based administrative data and hosting monthly meetings to review the quality of those data with district-level data staff. However, with the replacement of paper-based reporting for web-based reporting, some state-level respondents said that the state leadership felt a less prominent role for the data unit was justified. Regardless, district data staff and state-level respondents universally expressed the importance of establishing clear lines of reporting from district-level data units to the state-level data units. State-level respondents further articulated that building capacity for data analysis and data quality in the Directorate would be critical for improving accountability for data quality.

Fourth, some district-level staff explained the difficult trade-off they faced between upholding data quality or using data to show good district performance, because the latter factored into their performance assessment: ‘You can look at the data to actually see whether or not things are improving and to track health programs; or, you can focus on looking at the data mainly as a way to save your own job’ (I-13, District data staff). District-level staff also indicated that this conflict of interest was also applicable to community, block and district staff/officials.

Fifth, district-level staff explained how political or personal connections with members of the legislative assembly or district health and administrative officials weakened accountability for data quality at lower administrative levels. District data staff described how those who manipulate data at the community or block level were protected by district health officials who they had bribed for their current positions or were connected with through existing kick-back systems.

Finally, and related to the point above, influential connections coupled with high levels of discretion meant that senior block and district health officials rarely bore the consequences of engaging in corrupt practices like data manipulation. For example, a district data staff described being unable to enforce disciplinary measures against a block health official who was extorting a portion of the financial incentives given to community health workers for supporting institutional deliveries:

*The previous CMO would have called the MOIC [medical officer-in-charge] and said, ‘Listen I heard this news. You need to give me this much for this problem to go away…’* (I-29).

Politics aside, many district-level respondents pointed to the lengthy process for dismissing and suspending government employees (Legal Service India, 2018), which made holding them accountable very difficult. Despite being aware of the problems associated with data manipulation, district administrative officials submitted that in the light of significant human resources constraints, making do with the staff they had was their only option.

#### Enforcement of district-level data quality accountability mechanisms

District staff identified two factors contributing to the strong enforcement of data quality accountability mechanisms at the district level. First, district health and administrative officials who prioritized good quality data enforced the implementation of supportive supervision visits and reviewed the resulting reports. Second, many district health officials with better enforcement of data quality processes held weekly meetings with block-level officials and staff to ensure the consistent achievement of targets, troubleshoot problems and safeguard against data manipulation.

On a broader scale, there was a consensus across district-, division- and state-level respondents that the replacement of paper-based reporting with digital reporting increased accountability for good quality data and reduced opportunities to manipulate data at lower administrative tiers:

*Now with HMIS [the new web-based reporting platform], manual [paper-based] reporting is decreasing and things are improving. Before it was difficult to catch errors. Now with digitization, it’s easier for us to go back in time and see what data were being reported. With online data, we have capacity to do more analytical work.* (I-78, State-level respondent)

Interviews with division- and state-level respondents also signalled increasing prioritization of data quality at higher administrative levels with the recent development of a division-level monitoring and evaluation unit as well as a state-level data validation committee and audit team. State-level respondents also noted that demand for the states to improve data quality was coming from the national level, particularly, the Niti Aayog, India’s planning commission:

*Niti Aayog is not accepting [paper-based] data so all are focusing on HMIS data and that has to be correct and complete… they are ensuring HMIS should have correct data entry, data validation committee should be there, data should be checked and data output should be maximized, so this process is beginning now.* (I-55, State-level respondent)

### Pressures to manipulate data

#### Performance pressures

A high-pressure environment geared towards results and the achievement of targets was evident in the content and number of meetings held at the district level and between districts and the state. Many district staff described feeling ‘frustrated’, ‘burnt out’ and ‘overburdened’. Low achievement in the district rankings resulted in videoconferences with senior state-level leadership, which many district data staff described as a ‘one-way communication’ where the state leadership restated its performance expectations (I-25).

District staff felt this top-down pressure was reiterated during district-level meetings with district administrative officials. If achievements were lagging on certain priority programmes based on the district rankings, district administrators (e.g. district magistrates) would demand improvement. District staff universally stated that the district magistrates would never suggest ‘changing or manipulating the data’; however, to steer clear of their ‘scolding’ during the next meeting, district and block health officials would demand their staff to ‘increase the reporting’ of priority health indicators. As one district data staff explained:

*The thing is that they [block health officials and staff] have already understood that in the previous meeting, I was scolded for this. … So, if the MOIC was scolded for HBNC [home-based newborn care] last month; to take care of that next month they will do this [change the data]* (I-43).

#### Work culture: a punitive environment

Fear-based tactics, such as transfers or holding back salaries, were often employed by district health and administrative officials to increase accountability for programme performance. These tactics were observed during district-level meeting observations and corroborated by the district health and administrative officials we interviewed, who noted using similar approaches.

While a poor performance evaluation may be appropriate for poor performance, district staff felt these evaluations should recognize the effort and process, and not just outcomes. Many district staff described ‘holding their breath’ when attending district-level or state-level meetings where district rankings were reviewed, fearing repercussions for poor performance. The pressures to ‘fix’ or ‘improve’ performance fostered an organizational culture where data manipulation became a coping strategy, which was operationalized via informal networks with district and block health officials and data staff at the block and district levels. At times, top-ranking districts were also scrutinized for the suspicion of data manipulation. To draw less attention during meetings with district administrative officials or state-level officials, one district data staff said: ‘The CMO is satisfied as long as we are ranked somewhere in the middle [of the district rankings]. Same with DM [District Magistrate]’ (I-43).

### Rationalization

Not all district staff succumbed to the pressures of their environment. Many responded to these pressures by using existing technical reasons for being unable to manipulate data. One district data staff recalled refusing a request by clearly stating: ‘We [assistant research officers/district data staff] are not data generators. We compile data’ (I-37). Other district data staff described flatly ignoring requests to change data by refusing to ‘submit’ manipulated data on the HMIS web-based data portal. In another example, a district data staff described a peer, who continued to report actual attendance data of block-level medical officers despite facing high levels of pressure to inflate their attendance. However, persistently ignoring these requests resulted in a hostile work environment, which eventually led to the data staff’s resignation.

Data manipulation was seen as the way to survive within the broader system that appeared to condone the practice. District data staff described how their district health officials felt peer pressure to demonstrate progress when comparable districts had high district ranks, which they speculated was due to over-reporting (I-37). District data staff attributed quick spikes in monthly district rankings to the manipulation of district ranking indicators, as many district data staff stated, ‘there are rapid increases in district rankings even though these improvements should happen overtime and gradually’ (I-54). In this context, district staff noted that resisting data manipulation was particularly challenging. District-level staff also described how job security and financial security for one’s family were often used as justification for data manipulation, as one district data staff described forsaking his desire to ‘change the system’ to ensure that his ‘family is taken care of’ (I-13).

## Discussion

While data manipulation, such as the falsification of records for personal financial gain, and misreporting of administrative data to meet targets or to benefit from results-based aid programmes have been previously examined (Qazi and Ali, 2011; Sandefur and Glassman, 2015; Closser, 2022), there is a limited understanding of the underlying drivers of data manipulation. This study describes the main forms of data manipulation observed with administrative data and focuses on explaining the underlying factors that incentivize data manipulation, as well as the informal networks that operationalize its practice at local levels.

Notwithstanding the large investments in strengthening HMIS systems, these results highlight the importance of how the data are actually used in their organizational setting. Even though the goal of HMIS policies was to promote the use of good quality data in decision-making in the state, maintaining or achieving good district rankings took precedence over ensuring data quality mechanisms. We found competing systems of accountability at the local levels: district leadership enforced accountability for performance by reprimanding their staff for poor district rankings but rarely applied accountability for good data quality. Similarly, top-down pressures from the state level—partly due to weak state-level data units—resulted in limited demands for better data quality and increased accountability for performance data. We anticipated that respondents from high-priority districts, which received additional technical support, would experience fewer pressures to manipulate data. However, they reported similar experiences to respondents from non-high-priority districts. This suggests that data manipulation is not a mere reflection of insufficient resources but of broader organizational culture issues.

Performance pressures were reinforced by what was viewed as a punitive performance management system, where district staff and officials were named and shamed during meetings with their supervisors for low district rankings, resulting in a major conflict of interest. The performance of district/block health officials, who were responsible for data quality, was also judged on those same data via the district ranking. This situation perversely incentivized data manipulation, weakening the formal channels of accountability, transparency and enforcement of good data quality. Furthermore, because the job security of junior staff was tied to their obedience to superiors’ directives, the discretionary powers of officials who demanded data manipulation often remained unchecked. For some, data manipulation became a coping strategy to ‘improve’ performance over a short time period; as other officials observed such rapid improvements, they then used this to justify their own data manipulation.

George (2009) highlights that formal hierarchical mechanisms can be viewed as ‘a form of harassment’ and ‘dangerous’ where disciplinary actions may leave those lower in the organizational hierarchy feeling vulnerable and can foster opportunities for corruption and abuse (George, 2009). Qazi and Ali (2011) have also explained how top-down pressures have exacerbated data quality problems and resulted in the misuse of data for political gain. Similarly, Coutinho *et al*. (2000) described how manipulating data ‘to maintain a good record comes to eventually displace service to the clients as a primary goal’ in the context of immunization data (Coutinho *et al.*, 2000). In order to achieve targets and present a favourable image of the government, improving data to reflect good performance becomes the primary objective (Coutinho *et al.*, 2000). Demands and enforcement of accountability mechanisms by donors, politicians and senior officials have also contributed to the falsification of data, limiting the use of data and undermining the goal of building strong and functionable health information systems (Qazi and Ali, 2011; Storeng and Béhague, 2014). Some have argued that league tables, which are akin to district ranking dashboards, ‘create pressures for gaming measurement systems’, resulting in the development of ‘secret’ networks to counteract these pressures (Power, 2004).

In response to these challenges, there has been an effort to develop performance management approaches, such as balanced scorecards, that encompass broader domains, such as health worker satisfaction, patient satisfaction and staff knowledge, which are critical to performance management (Peters *et al.*, 2007). Metrics that capture these domains are expected to provide a holistic perspective on performance, thereby alleviating pressures to game the system.

In this Indian state, while the development of the district rankings helped create a culture of accountability, it also led to unintended consequences of placing blaming and punishing staff, as well as data manipulation. Vian’s conceptual framework offers a guide to developing strategies to mitigate data manipulation in this context. To reduce opportunities to manipulate data, first, the incentive structures need to change so that there is equal emphasis on data quality as on performance data. For example, HMIS architects may consider ‘randomizing’ the selection of indicators used to compute the district rankings. Additionally, requiring district and state officials to review the data quality results from supportive supervision visits and data validation committee meetings, alongside the review of the monthly district rankings, may also incentivize data quality.

Second, strengthening checks and balances can help reinforce the integrity of data-related processes at all levels. Such strategies to increase checks and reduce pressures to manipulate data may include computing a metric for data accuracy, which is integrated into the monthly district rankings, and implementing a ‘naming and faming’ strategy (Zomer, 2018) that recognizes blocks and districts that show excellent data quality measures. Expanding the pool of district ranking indicators to include measures of inputs and processes that influence the performance of health services may also encourage state-level decision-makers to understand strengths and deficiencies affecting the achievement of targets for health services. More broadly, strategies that enable district teams to achieve targets without having to resort to data manipulation may include (1) establishing or strengthening district- or division-level technical units that can provide targeted guidance to districts on how to improve the implementation and performance of their health programmes and (2) ensuring that these units have access to the relevant resources and skill sets necessary to achieve the intended goals. Furthermore, the digitization of HMIS also provides opportunities to streamline data collection and entry and implement automated data validation checks, which can help facilitate data audits and contribute to improved data quality (Neupane *et al.*, 2014; Charanthimath *et al.*, 2021).

Third and finally, implementing system-wide policies that make data manipulation an unacceptable anomaly will be critical for discouraging the rationalization of data manipulation. This could involve articulating the consequences of data manipulation in policy guidelines, and devising strategies to make the practice less socially acceptable would be critical for promoting a shift in attitudes.

Our study is not without limitations. First, we were unable to extensively capture the views of district magistrates whose insights might have further illuminated the findings from this study. We also did not examine the views of facility-level staff who are responsible for data entry at the block and community levels and might have offered further insights into the drivers of data manipulation at these levels. Third, we recognize potential social desirability bias among respondents; however, through prolonged engagement and rapport building, we attempted to address this limitation and generate a detailed and nuanced analysis. Finally, this study was not designed to quantify the frequency and magnitude of data manipulation.

## Conclusion

This study unpacks the main drivers of data manipulation and shows why these practices persist despite the strong initiatives to improve data quality by the government. A deeper understanding of the underlying barriers to data manipulation is the first step towards identifying strategies to curb this practice. While stakeholder engagements will be required to identify context-appropriate strategies in this Indian state, our study identifies three main entry points to mitigate data manipulation: (1) changing the incentive structures, for there to be equal emphasis on data quality as there is on performance data, (2) strengthening checks and balances to reinforce the integrity of data-related processes at all levels and (3) implementing system-wide policies that make data manipulation an unacceptable anomaly. Future evidence on context-relevant top-down and bottom-up strategies to effectively counter data manipulation is required.

## Data Availability

The underlying data for this manuscript cannot be shared publicly for ethical and privacy reasons. Sharing these data would undermine the informed consent process, which did not indicate that these data would be shared publically. In addition, these data contain sensitive information that could reveal identifiable information about the participants, their roles and responsibilities and their employers. Requests to access anonymized data will need to be made to the corresponding author and the relevant Institutional Research Committees that oversaw this study.

## Reflexivity statement

The authors include three females and one male and span multiple levels of seniority. All the authors have worked extensively in India, particularly in the state where the research was conducted. The author who conducted the interviews is of Indian descent and fluent in the local language spoken in the state. All the authors collectively have experience and expertise with qualitative methods, health systems research and issues around data use for decision-making.

## Appendix

**Table A1.** Categories and sub-categories in the analytical framework

Categories	Sub-categories
Rationalization of data manipulation	Perceptions of being empowered in the workplace autonomy self-efficacy ability to make a difference meaning or commitment to one’s work Morality/ethics Social norms
Opportunities to manipulate data	Discretion Level Controls Accountability for performance Measurement of goals Monitoring mechanisms Sanctions/consequences Accountability for data quality Measurement of goals Monitoring mechanisms Sanctions/consequences
Pressures to manipulate data	Pressures Performance Fear-based (job security, withholding pay and scolding) Workload (including time pressure)
Other factors	[Open-ended]
Types of data manipulation	Types of data Level Participants/network
